# Real-Time Detection of Tumor Cells during Capture on a Filter Element Significantly Enhancing Detection Rate

**DOI:** 10.3390/bios11090312

**Published:** 2021-09-03

**Authors:** Astrid Lux, Hannah Bott, Nisar Peter Malek, Roland Zengerle, Tanja Maucher, Jochen Hoffmann

**Affiliations:** 1 Robert Bosch GmbH, Corporate Sector Research and Advanced Engineering, Robert-Bosch-Campus 1, 71272 Renningen, Germany; hannah_bott@outlook.de (H.B.); Tanja.Maucher@de.bosch.com (T.M.); Jochen.Hoffmann2@de.bosch.com (J.H.); 2 Department of Internal Medicine, University of Tübingen, Otfried-Müller-Str. 10, 72076 Tübingen, Germany; Nisar.Malek@med.uni-tuebingen.de; 3 Laboratory for MEMS Applications, Department of Microsystems Engineering, University of Freiburg, Georges-Koehler-Allee 103, 79110 Freiburg, Germany; zengerle@imtek.uni-freiburg.de

**Keywords:** liquid biopsy, circulating tumor cells, filtration, size-based enrichment, immunocytochemistry, real-time detection, lab-on-a-chip

## Abstract

Circulating tumor cells (CTCs) that enter the bloodstream play an important role in the formation of metastases. The prognostic significance of CTCs as biomarkers obtained from liquid biopsies is intensively investigated and requires accurate methods for quantification. The purpose of this study was the capture of CTCs on an optically accessible surface for real-time quantification. A filtration device was fabricated from a transparent material so that capturing of cells could be observed microscopically. Blood samples were spiked with stained tumor cells and the sample was filtrated using a porous structure with pore sizes of 7.4 µm. The possible removal of lysed erythrocytes and the retention of CTCs were assessed. The filtration process was observed in real-time using fluorescence microscopy, whereby arriving cells were counted in order to determine the number of CTCs present in the blood. Through optimization of the microfluidic channel design, the cell retention rate could be increased by 13% (from 76% ± 7% to 89% ± 5%). Providing the possibility for real-time detection significantly improved quantification efficiency even for the smallest cells evaluated. While end-point evaluation resulted in a detection rate of 63% ± 3% of the spiked cells, real-time evaluation led to an increase of 21% to 84% ± 4%. The established protocol provides an advantageous and efficient method for integration of fully automated sample preparation and CTC quantification into a lab-on-a-chip system.

## 1. Introduction

Circulating tumor cells, which are shed from the primary tumor into the vasculature, are described as the major cause for metastases [1]. These cells have been found in the blood of breast, lung, prostate, and colon cancer patients and have been correlated to poorer outcome and tumor aggressiveness [2,3,4,5]. For quantification of the cells that reside in the blood despite therapy, the use of liquid biopsy provides a minimally invasive method for therapy monitoring and personalized treatment optimization [2]. A decrease in the number of CTCs seems to correlate to treatment response, whereas an increase might require adaptation of the therapy [6]. Besides the enumeration, phenotyping, cultivation of spheroids and organoids, and genotyping thereof is possible after isolation of CTCs and could provide valuable information for therapy adaptation [7]. However, due to the rarity and heterogeneity of the CTCs, their enumeration is a huge technological challenge. The exact number in patient samples is not known and can differ both between different patients and even within one patient during therapy [8]. Thus, the definition and application of a positive control is difficult and represents an additional challenge during assay development. Generally accepted controls are blood samples from healthy donors in which no CTCs are detected as negative control altogether with high recovery of cell line cells spiked into healthy blood as positive control [9]. CTCs can be isolated from blood based on their biological and physical properties, as for example characteristic surface proteins, or their size, stiffness, and density [10,11,12,13,14]. One major challenge in CTC isolation is the high number of background cells such as leukocytes and erythrocytes that need to be depleted to enable optical detection of the CTCs on one hand and ensure sufficient sensitivity of the assay in the other hand. So far, the only FDA-approved and most clinically-validated CTC isolation and detection method is the CellSearch^®^ method. Hereby, the cells are immunomagentically enriched using EpCAM-coated ferrofluid nanoparticles and afterwards the enriched cell are evaluated by immunostaining of Cytokeratins (CK). However, only very few CTCs are isolated by this method and sensitivity varies greatly [3,15]. In order to increase reproducibility of sample preparation and enable CTC detection at the point-of-care, several LoC-based methods for isolation and quantification were established and extensively studied as reviewed in [16,17]. Such LoC systems enable microfluidic processing of samples for separation of different cell fractions and isolation of target cells from blood based on immunoaffinity [18] centrifugo-magnetophoretic [19], dielectrophoretic methods [20], filtration [21,22], or a combination thereof [23].

In 1964, Seal first described a dead-end microfiltration approach for CTC isolation from whole blood as CTCs were found to be larger in terms of size and lower in terms of deformability than white blood cells and erythrocytes [24] Advantages of filtration approaches for cell separation are high efficiency, low fabrication costs, simple hands-on steps, the possibility to combine isolation with downstream analysis, and short processing time. Mechanical filtration approaches do not rely on the expression of epithelial markers of the CTCs and are considered as label-free methods [25]. The criteria for filtration-based cell selection is their size and deformability. Thus, also cells that underwent epithelial-to-mesenchymal transition (EMT) are enriched. These cells are described to be the critical CTC population during metastasis formation. Moreover, mechanical filtration was proven to be relatively robust to flow rate variations, making them a suitable method for integration into pneumatically-driven, membrane-based LoCs like the Vivalytic system from Bosch Healthcare Solutions GmbH [26]. Parallel enrichment of other large blood cells like megacaryocytes, large monocytes, epithelial cells from the puncture site, and mesenchymal cells entails the challenge of establishing a suitable recognition method, for instance, by immunocytochemistry. The following requirements have been described for the clear distinction of the different cells enriched on a filter: cells counted as CTCs need to be epithelial cell adhesion molecule (EpCAM) positive, CD-45-negative, viable, and contain an intact nucleus [27]. Filtration-based methods are prone to enrich false positive cells while losing rare CTCs that are very small (<8 µm) or cells that are very deformable [28]. Clogging of the filter can also be a challenge of filtration-based approaches.

Most methods described in literature for isolation of CTCs by filtration-based approaches, detect cells after completion of the filtration process. Often, filters need to be removed from the filtration device for subsequent microscopic analysis [29,30]. Besides, several methods describe that cells are stained after filtration. Low capture rates, loss of cells and clogging of the filter can be overcome by a larger filter surface with higher pore numbers. However, this requires x,y-scanning of the filter surface and thus end-point imaging. Thus, cells that passed the filter during the process are not detected. Quantification efficiency can be improved by integration of real time detection during the process of CTC isolation, so that cells are detected and counted as they pass the detection system as for example in a modular microsystem described in [31] that enables both label-independent impedance-based counting and phenotyping by immuno-staining. Another example is the Parsortix™ system, in which the cells are sorted by a filtration casette and the cells are stained automatically. The stained cells can be detected by placing the casette under a fluorescence micrsocope. However, the area of the casette is too large to image the total area at once [32].

In this study, we present a method for the CTC capture on a porous structure and the advantages of real-time detection during the filtration process for coping with the challenge of missing small and deformable cells in a filtration approach with end-point optical detection of the captured cells after the filtration process. The aim of this work was to develop a method for real-time monitoring of a CTC filtration process compatible with the Vivalytic system. The Vivalytic system is a membrane-based microfluidic network consisting of microfulidic channels and functional units actuated by a peristaltic pump. Reagent storage and fully automated sample processing like cell lysis and staining are possible after introduction of the sample into the system. For automatization, the Vivalytic system would enable the CTC detection in a sample-to-answer way through processing a whole blood sample including all sample preparation steps (blood lysis, staining, filtration, and detection). This approach provides two key advantages: automation of CTC isolation with minimal hands-on preparation and the possibility to observe and count the cells in real-time as they arrive on the filter surface. This maximizes the quantification efficiency, even though cells might pass the filter after some time during the process. We investigated the capture rates for different cell types on a porous structure and to the best of our knowledge, this is the first study evaluating the increase of detected cells when capture was observed in real-time with the focus on counting rather than recovering all CTCs present in a sample for downstream analysis.

## 2. Materials and Methods

### 2.1. Blood Samples

Blood samples from pseudonymized donors (Ethics statement project number 480/2019BO2; University Hospital Tübingen) were collected in 3.4 mL or 7.5 mL EDTA tubes (Sarstedt, Nümbrecht, Germany) and stored at 4 °C for a maximum of 3 days. The samples were spiked with different cell lines in order to obtain model blood samples containing known numbers of target cells. 

### 2.2. Cell Culture and Sample Preparation

Human breast cancer cell line BT-474 was obtained from the DKFZ (Deutsches Krebsforschungszentrum), and human colon cancer cell line HCT116 and hematopoietic cell line K-562 were obtained from the Leibniz Institute DSMZ. Cell lines were routinely cultured in DMEM medium supplemented with 10% fetal serum albumin and 1% Pen/Strep under standard conditions (37 °C, 5% CO_2_, 100% humidity). Cells were harvested by trypsination for 3–5 min at room temperature. Cell passages from 4 to 30 were used for experiments. Cell spiking was performed by first diluting the cell suspension to 3–6 × 10^3^ cells/mL and staining the cells with Carboxyfluorescein succimidyl ester (CFSE; Thermo Fisher Scientific, Waltham, MA, USA) following the manufacturers’ protocol. Samples were incubated for 10 min. Three aliquots of 100 µL were transferred into a 96-well microtiter plate and cells were counted using an inverted fluorescence microscope (Olympus IX83, Olympus, Tokyo, Japan) and the CellSens Dimensions Software. In parallel, 100 µL aliquots were transferred into sample tubes for spiking into blood. The mean cell number of the three aliquots was defined as cell spike number. Prior to filtration, the erythrocytes in the blood were selectively lysed by the addition of four volumes of erythrocyte lysis buffer (Qiagen, Hilden, Germany).

### 2.3. Immunocytochemistry

Spiked cell line cells were stained with either CFSE (Thermo Fisher Scientific, Waltham, MA, USA; Ex/Em 492/517) in 1× phosphate buffered saline (PBS), or EpCAM-specific antibodies conjugated to fluorescein isothiocyanat (FITC; Ex/Em 492/517; Miltenyi Biotec, Bergisch Gladbach, Germany) at 1:50 dilution (final concentration of 0.4 µg/mL) in PBS with 1% (w/v) bovine serum albumin (BSA) and 1 mM ethylenediaminetetraacetic acid (EDTA). Leukocytes were stained with anti-CD45-PE at 1:50 dilution (final concentration of 0.4 µg/mL; phycoerythrin Ex/Em 565/578) in whole blood. In order to prevent non-specific staining, whole blood and cell line cells were stained separately before spiking into blood. Direct staining of the spiked cells by addition into the blood and spiked cell suspension was also performed for assay verification. However, in this study, for the proof-of-principle and determination of the cell capture and detection on the filter, it was important to stain the cells separately. 

### 2.4. Determination of the Cell Capture Rate and Recovery 

**Figure 1:**
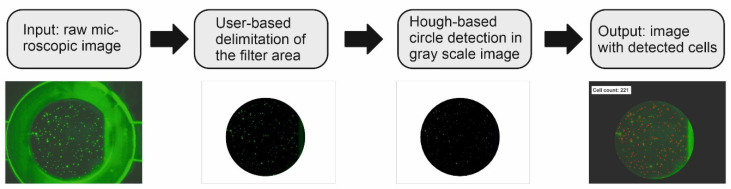
Framework of the digital image processing for cell recognition with circular Hough transform-based algorithm: Step (1) Read in the microscopic fluorescence image; Step (2) User-based determination of the circular filter section. Fluorescent edge regions are cut off to minimize interfering signals in the image for cell detection; and Step (3) Circular Hough gradient transform is applied for cell recognition. Position and radius of every detected cell are stored in an array. Step (4) Generation of output image with detected cell count and positions.

The effective filter area of the TEM-grid with a diameter of 2 mm resulting in an area of 3.14 mm^2^ was completely visible by fluorescence microscopy at 50-fold magnification during the filtration process (Figure 1). Thus, the arrival of the cells of the filter could be observed in real-time. By serial or video recording, both captured and passing cells were detected at a certain time point. Digital images in the respective fluorescence channels (EGFP, Cy3 filter sets, Olympus, Tokyo, Japan) were acquired with an Olympus BX63 epi-fluorescence microscope (Olympus, Tokyo, Japan) with CCD camera (C4742–95, Hamamatsu, Hamamatsu, Japan) and a 5×/NA0.13 objective (Olympus, Tokyo, Japan).

The number of stained cells was enumerated semi-automatically using the microscope software cellSens Dimension Software (Olympus, Tokyo, Japan) and an algorithm developed in Matlab (Mathworks, Natick, MA, USA). For end-point quantification, the cells on the filter were counted using the *Count and Measure* solution module included in the cellSens Dimension Software (Olympus, Tokyo, Japan). By selecting a region of interest (ROI) and setting a manual threshold, the cells were identified based on their fluorescence intensity and included into the count based on predefined settings for the minimum radius (based on the spike cell line size), the shape, and sphericity factors of the detected objects in the region of interest.

Capture rate was defined as the percentage of spiked cells identified on the filter after the whole filtration process (end-point). Recovery rate was defined as the percentage of spiked cells that were detected in the field of view during the whole filtration process in real-time. Moreover, cells that have passed the filter were collected and counted to ensure that no cells were lost inside the microfluidic system.

(1) $$\mathrm{Capture}\;{\mathrm{rate}}_{\mathrm{end}-\mathrm{point}}\;[\%]=\frac{\mathrm{number}\;\mathrm{of}\;\mathrm{cells}\;\mathrm{captured}\;\mathrm{on}\;\mathrm{the}\;\mathrm{filter}\;\mathrm{after}\;\mathrm{filtration}}{\mathrm{number}\;\mathrm{of}\;\mathrm{cells}\;\mathrm{spiked}\;\mathrm{into}\;\mathrm{the}\;\mathrm{sample}}\times 100$$

(2) $$\mathrm{Recovery}\;{\mathrm{rate}}_{\mathrm{real}-\mathrm{time}}\;[\%]=\frac{\mathrm{number}\;\mathrm{of}\;\mathrm{cells}\;\mathrm{observed}\;\mathrm{above}\;\mathrm{the}\;\mathrm{filter}\;\mathrm{during}\;\mathrm{filtration}}{\mathrm{number}\;\mathrm{of}\;\mathrm{cells}\;\mathrm{spiked}\;\mathrm{into}\;\mathrm{the}\;\mathrm{sample}}\times 100$$

Besides filtration of cell line samples and spiked whole blood samples, the same samples were introduced into a set-up without TEM-grid integrated in order to evaluate whether cells are retained in the channels and connection sites of the microfluidic structure. Therefore, a defined number of cells was drawn through the set-up at 2 µL/s and afterwards the cells in the outlet suspension were counted by fluorescence microscopy.

### 2.5. Matlab Evaluation

To determine the number of target cells present in the sample/retained on the filter, a Matlab-based code was developed based on the circular Hough gradient transformation as alternative for the cellSens Dimension software module. The general framework for the image processing is displayed in Figure 1.

The Matlab code for cell recognition was extended to evaluate image sequences of an entire filtration process. The framework for image sequence processing now included reading in a sequence of images, determining the number positions of cells for each image in the sequence, and finally comparing all determined positions over time to eliminate double-counted cells. Again, the method was evaluated by comparing the result to a manual cell count over time and the result obtained by the cellSens Dimension module.

### 2.6. Microfluidic Filtration Device and Method

#### 2.6.1. Fabrication of the Cell Capture Device

**Figure 2:**
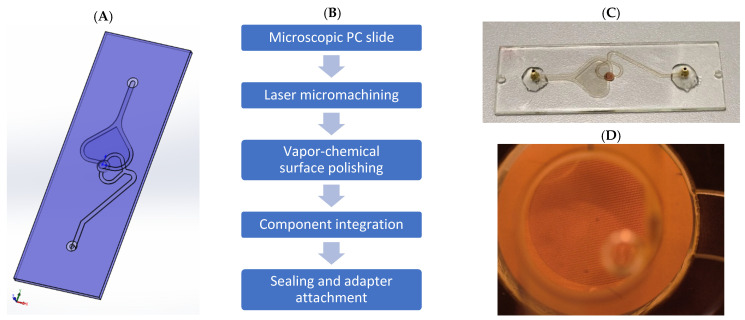
Design and fabrication steps of the cell capture device. (**A**) CAD design of a microscopic PC slide. Coordinates for laser micromachining were imported from STEP file. (**B**) Fabrication steps for PC slide to obtain cell capture device. The CAD design was machined into a PC slide through laser ablation. After vapor-chemical surface polishing and smoothing, the filtration component was integrated using an adhesive. After sealing of the microfluidic structures through sealing foil and adaption of tube adapters, the slide was ready to use. (**C**) Fabricated PC slide as cell capture device with integrated TEM-grid as filtration component. (**D**) Microscopic image of mounted TEM-grid.

The cell capture device was designed for and implemented into injection-molded polycarbonate (PC) slides with a size of 75.5 mm × 25.5 mm × 1.5 mm. Figure 2 gives an overview of the steps involved in the fabrication process: First, a computer-aided design (CAD) layout of the filtration unit with inlet and outlet channels was designed. The designed microfluidic structures were then micromachined into the PC slides by ultrashort pulse laser ablation (Nd:YAG laser, Lumera GmbH, Kaiserslautern, Germany; Laser scanning optics and CNC stage, GFH GmbH, Deggendorf, Germany). After laser micromachining, the surface of the polymer parts was polished by a vapor-chemical treatment as it was developed in [33], to improve the optical transmittance of the filtration unit and enable a sufficient sealing with the sealing foils. Before the sealing of the microfluidic structures, the filter component (e.g., a Transmission Electron Microscope (TEM)-grid, see next section) was implemented into the device through a microdispenser via adhesive bonding (UHU plus sofortfest 2 Komponenten-Kleber, UHU, Bühl/Baden Germany).

The structures were sealed with an adhesive film (MicroAmp™ Optical Adhesive Film, Thermo Fisher, Waltham, MA, USA) and tube adapters were attached to the inlet and outlet of the device via adhesive bonding (UHU plus *schnellfest* 2 *Komponenten-Kleber*, UHU, Bühl, Germany). After a curing time of 12 h, the device was ready for use.

#### 2.6.2. Integration of Filtration Component

A TEM-grid was integrated into the cell capture device for filtration and capture and detection of cells in suspension or spiked into blood samples. They were purchased from Plano GmbH. The TEM-grids had a nominal pore size (S) of 6.5 µm and a bar width of 6 µm between the pores. Pore pitch (C) was specified as 12.5 µm and porosity as 41%. Microscopic examination (image shown in supplementary Figure S1) did not confirm the specifications and revealed a pore size (S) of 7.44 ± 0.06 µm and a bar width of 4.5 µm. Porosity (P) was determined using the following equation for an area with round holes in hexagonal structure:

(3) $$P\;\left[\%\right]=\frac{S^{2}\times 0.9069}{C^{2}}\times 100\%$$

resulting in a porosity of 35.2%. The TEM-grid had an area of 2.6 mm^2^ and 17,000 pores. To obtain a cell capture device, the TEM-grid was mounted into the PC slide. Adhesive bonding ensured the fixation and sealing of the integrated filter.

#### 2.6.3. Filtration Procedure

**Figure 3:**
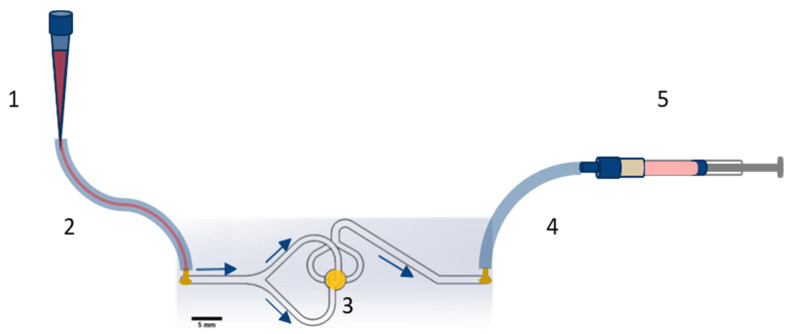
Scheme of the filtration setup with its components. The syringe was operated by a syringe pump. To operate the filtration setup, the cell containing sample was filled into the pipette tip, serving as inlet funnel (1) connected to the set-up via silicone tubes (2 and 4), and drawn over the filter (3) into the syringe (5) operated by a syringe pump (not shown in the image) with a defined flow rate profile. The cells captured on the TEM-grid were detected by fluorescence microscopy at 50-fold magnification.

Before filtration of a sample, the set-up shown in Figure 3 was primed with 1 × PBS/1% (*w/v*) BSA/1 mM EDTA and incubated for 30 min at room temperature. Afterwards, the cell line sample or spiked blood sample was drawn over the TEM-grid at variable flow rates, driven by a syringe pump (Low Pressure Syringe Pump neMESYS, Cetoni GmbH, Korbußen, Germany), and the filtration process (as explained in the Supplementary material, Figure S2) was observed by fluorescence microscopy (set-up shown in Figure S3). The sample drawing was followed up by a washing step with 1 × PBS/1% (*w/v*) BSA/ 1 mM EDTA of the same volume to rinse away residual antibody/dye, hemoglobin, and lysed erythrocytes.

#### 2.6.4. Statistical Analysis

Data are presented as means ± standard deviation of the mean. Statistical analysis was performed using SPSS statistics software version 25 (IBM, Armonk, NY, USA). Shapiro–Wilk’s test and Levene’s test (*p* < 0.05) were applied to evaluate normal distribution and homogeneity of variances. Differences between groups were determined using t-test or one way ANOVA for normally distributed data sets with homogeneous groups variances, Welch’s test with the Dunnett-T3 posthoc test for normally distributed data sets with heterogeneous group variances, or Kruskall–Wallis test for data sets that are not normally distributed. *p* < 0.05 was considered to indicate significant difference. Significant differences were displayed by different letters above the bars.

## 3. Results

### 3.1. Identification of a Robust Operation Set Up for Cell Detection and Capture Rate Determination

#### 3.1.1. Design of the Filtration Unit

In this study, the cell capture devices were produced with PC slides using the rapid prototyping method presented in the previous chapter. However, the device was designed for integration into the microfluidic cartridge of the LoC system *Vivalytic*. Therefore, it was intended to meet the manufacturing requirements of the LoC cartridge. These include manufacturing the PC components as injection-molded parts, as well as assembling and sealing the components in a multi-step laser-welding process.

**Figure 4:**
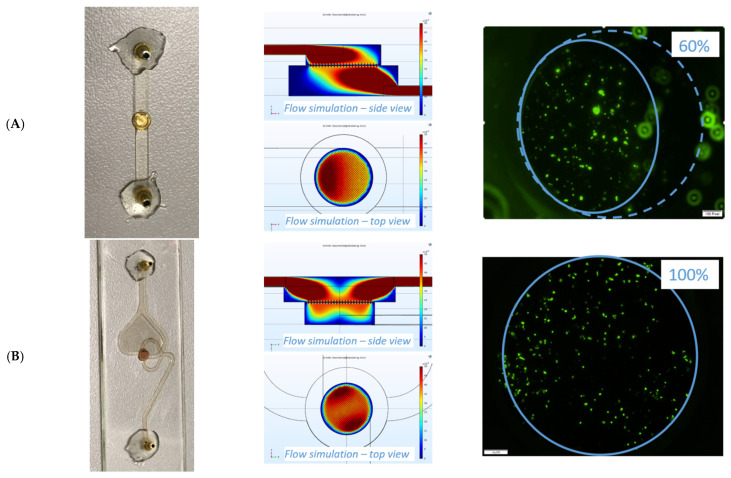
Design, simulation, and experimental determination of flow through area for cell retention on two filter holder designs. Design (**A**) contained a single inlet and outlet channel on the top and bottom of the slide. A flow rate of 2 µL/s resulted in a flow through area of ~60%. For the adapted design (**B**), the inlet was divided into two strands, which reach the filter the opposite way and slightly offset. Furthermore, the outlet was divided into two strands leaving the filter at 90 degrees from the inlets. A flow rate of 2 µL/s resulted in a flow through area of close to 100%.

In the first approach, a filter holder was designed in which the TEM grid could be integrated as a cell filter component, containing a single inlet and outlet channel on the top and bottom of the slide (see Figure 4A). A channel height of 400 µm was chosen according to the channel dimensions on the *Vivalytic* cartridge. Static fluid simulations of the filter holder were performed with the finite element-based simulation software COMSOL Multiphysics (version 5.5) and revealed that dividing both the inlet and the outlet into two strands improved the flow through area of the filter. The design of the filter holder was adapted dividing the inlet into two strands, which reach the filter the opposite way and slightly offset. Furthermore, the outlet was divided into two strands leaving the filter at 90 degrees from the inlets (see Figure 4B). Static fluid simulations of the filter holder revealed that the adapted design led to a more homogenous flow through the filtration area. The improvement of the flow through the filter was also investigated experimentally. Figure 4 shows the result after filtration of CFSE-labeled BT-474 cells at a flow rate of 2 µL/s. The area with retained cells increased from 60% to nearly 100% of the filtration area.

**Figure 5:**
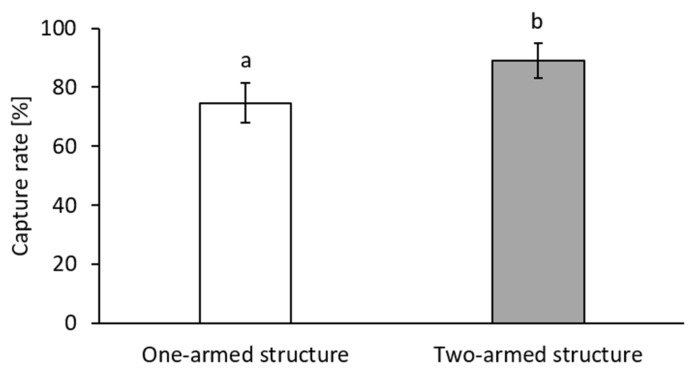
Capture rates of BT-474 cells spiked into 200 µL of blood and drawn through the TEM-grids integrated into the different filter holder slides. The capture rate was significantly increased by the filter holder design with the divided inlet and outlet channels. Results are given as means ± SD. Different letters above the bars (a and b) indicate statistical difference (*n* = 3; *p* < 0.05).

As shown in Figure 5, the improved flow through also led to improved capture rate, increasing from 76% ± 7% capture rate to 89% ± 5% capture rate for BT-474 cells.

#### 3.1.2. Distinction of Target Cells from Leukocytes

**Figure 6:**
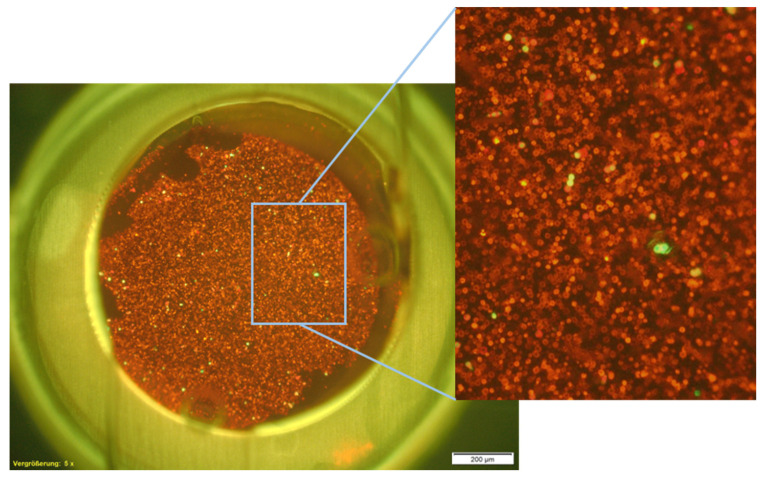
Leukocytes stained with anti-CD45-PE (orange) and spiked HCT116 cells stained with anti-EpCAM-FITC (green) captured on the TEM-grid. Detected by fluorescence microscopy at 50-fold magnification and 200 ms illumination time. The images were recorded separately in the respective channel and overlaid afterwards.

Target cells for spiking of the blood samples were either stained with CFSE or a labelled EpCAM antibody (anti-EpCAM-FITC, Miltenyi Biotech, Bergisch Gladbach, Germany). Figure 6 shows the membrane EpCAM staining of HCT116 cells and CD-45 staining of leukocytes captured on the TEM-grid (anti-EpCAM-FITC and anit-CD-45-PE, Miltenyi Biotech, Bergisch Gladbach, Germany). Images were recorded in the respective channels for each fluorescent dye and were overlaid afterwards for distinction of spiked cell line cells and leukocytes. In order to ensure that no EpCAM-positiv cells were covered by leukocytes, the filter area was scanned in different focal planes (z-positions). This revealed a monolayer of leukocytes, in which all EpCAM-positive cells were visible in one focal plane. Staining of additional surface antigens, such as cytokeratine, using different antibodies, would be possible in order to detect cells with low EpCAM expression and ensure clear distinction between CTCs and leukocytes that could be falsely stained due to unspecific binding of antibodies. However, in this study, we focused on examining the optimization of the detection rate based on real-time detection in comparison to end-point detection using cells with known homogenous EpCAM-expression as model antigen.

#### 3.1.3. Spike-and-Capture Linearity

**Figure 7:**
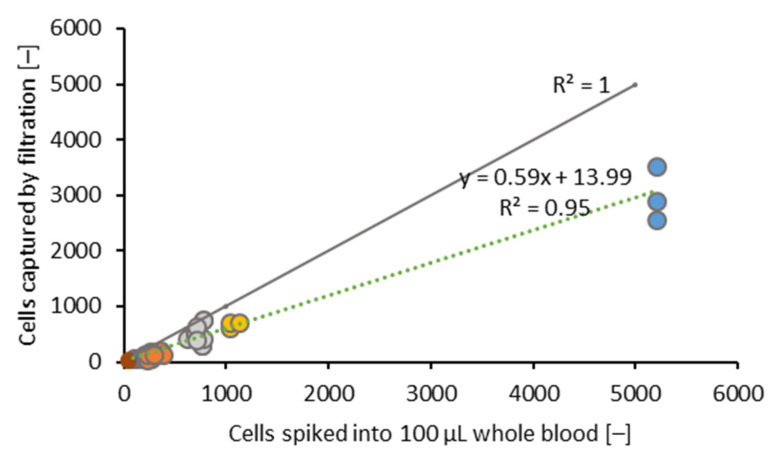
Linearity of the cell capture for 7.4 µm TEM-grids. Two hundred microliters of healthy whole blood were spiked with 50, 100, 300, 600, 1000, or 6000 cells. Filtrations for each cell number were performed at least in triplicates. The cells for spiking were stained and counted in 96-well microtiter plates by fluorescence microscopy beforehand. The grey line shows the spike-and-capture for a theoretical capture rate of 100%. The dotted line represents the linear fit of the spike-and-capture linearity of the HCT116 cells on the TEM-grid.

The number of spiked HCT116 cells plotted against the number of captured HCT116 cells is shown in Figure 7. A capture rate of 59% ± 14% was comparable (R^2^ = 0.95) for cell numbers of 50, 100, 300, 600, 1000, and 5000 HCT116 cells per 100 µL blood and is shown in Figure 7.

### 3.2. Parameters Influencing the Cell Capture Rate

#### 3.2.1. Effect of the Cell Size on the Capture Rate

**Figure 8:**
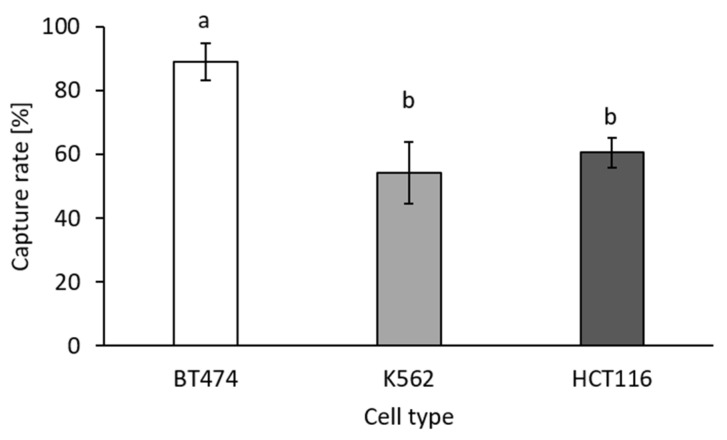
Capture rates for cell lines BT-474 (*n* = 5), K562 (*n* = 4), and HCT116 (*n* = 10) spiked into whole blood samples and filtration at a constant flow rate of −2 µL/s as determined by end-point detection. Results are given as means ± SD. Different letters (a and b) above the bars indicate statistical significance, results with the same letters (b and b) are not significantly different (*p* < 0.05).

Cancer cell line cells of different sizes were used to evaluate the filter system regarding the size-dependent cell capture rate. Breast cancer cell line BT-474 had an average diameter of 20 µm, hematopoetic cell line K562 had an average diameter of 19 µm, and colon cancer cell line HCT116 of 15 µm at the time of filtration. The capture rates for the three cell lines spiked into 200 µL of healthy whole blood are shown in Figure 8. As expected, the highest capture rate of 89% ± 5% was achieved for BT-474. The lowest capture rate of 54% ± 9% was achieved for the hematopoietic cell line K562. The latter cell line was used as non-epithelial EpCAM-negative control and was assumed to be more deformable than the other two cells lines due to its origin from human blood [34]. Therefore, a lower capture rate was achieved for this cell line despite the cells being larger than the HCT116. In order to challenge the filter system with small EpCAM-positive cells, the HCT116 cells were used for all further experiments. For these cells, an initial capture rate of 64% ± 5% was determined by end-point detection.

#### 3.2.2. Effect of the Blood Volume on the Capture Rate

Defined numbers of HCT116 cells were spiked into different volumes of blood and into 1 × PBS/1% BSA/1 mM EDTA buffer to determine the influence of the duration of filtration and proportion of contained leukocytes on the CTC capture rate. 

**Figure 9:**
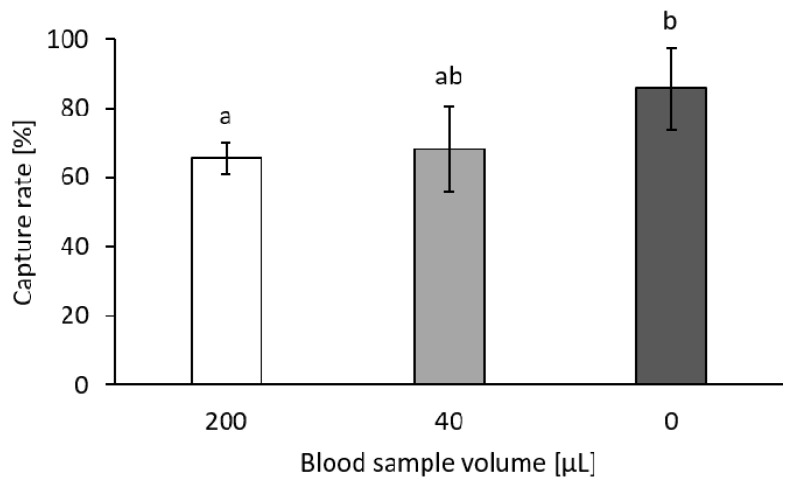
HCT116 cell capture rate dependent on the whole blood sample volume. Stained cells were spiked into 200 µL and 40 µL whole blood or 1 × PBS/1% BSA/1 mM EDTA as control. Results are given as means ± SD. Different letters (a and b) above the bars indicate statistical difference (*n* = 5; *p* < 0.05).

Figure 9 shows that the capture rate of the HCT116 cells significantly increased when cells were filtrated without the presence of blood, leading to the assumption that the increased processing time resulting from the higher sample volume and pressure increase caused by the leukocytes and erythrocytes present in a blood sample led to significant cell loss. The retained leukocytes caused clogging of pores and thus, the remaining cells needed to pass through pores that were not clogged. The more pores were clogged by leukocytes, the higher the pressure difference at open pores. In these areas, the flow rate increased and the cell could be pushed through the filter more easily [35]. However, there was no significant difference between a 40 µL blood sample and a 200 µL blood sample. Thus, the application of a higher blood sample volume in order to increase the probability and reliability of CTC capture does not influence the capture rate negatively.

#### 3.2.3. Optimization of the CTC Quantification by Real-Time Detection

**Figure 10:**
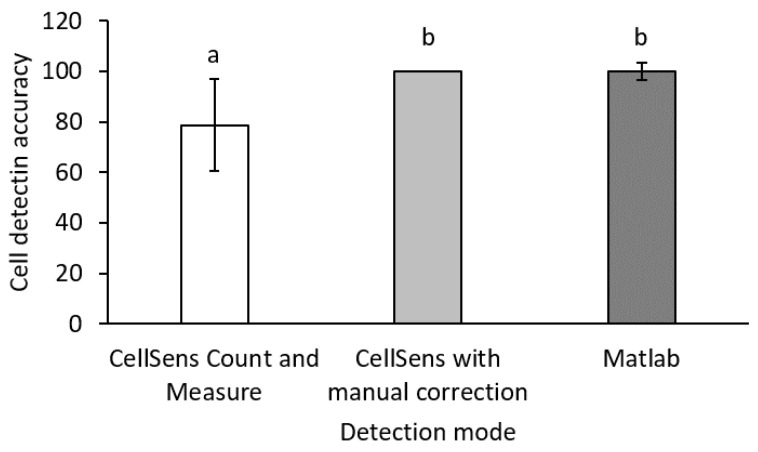
Comparison of the cell detection accuracy of the CellSens Count and Measure solution (Olympus, Tokyo, Japan) with and without manual correction and the custom Matlab-based evaluation. The results of the CellSens detection with manual correction were regarded as 100% reference. Results are given as means ± SD. Different letters above the bars (a and b) indicate statistical difference (*n* = 8; *p* < 0.05).

In order to enable automated cell detection, a Matlab-based method was established and compared to the Count and Measure Solution of the CellSens software. Hereby, the stained cells were imaged in the respective fluorescence channel, and afterwards an adaptive or manual threshold could be set so that all cells with a fluorescence intensity above the set threshold could be counted in the integrated Count and Measure Solution. Additionally, a different filter for sphericity and minimal diameter was set in order to exclude artefacts from the count. The CellSens-based evaluation was accurate when the threshold, filters for cell sizes, and sphericity were set accordingly. However, as seen in Figure 10, manual correction was necessary to count single cells in aggregates or in close proximity to each other. As seen in Figure S5, the Matlab-based evaluation was more accurate in the recognition of single cells within an aggregate. Altogether, using the Matlab framework of the digital image processing for cell recognition, comparable results were obtained as in the manually corrected CellSens-based evaluation that was regarded as reference at 100% (see Figure 10).

**Figure 11:**
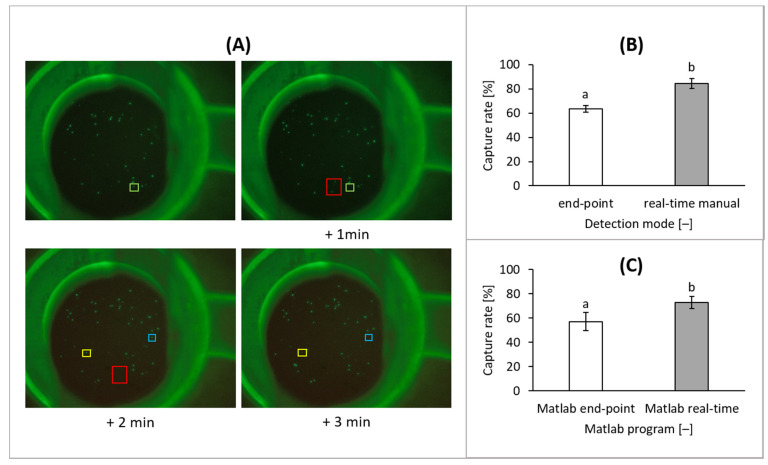
CFSE-stained cells in 1 × PBS/1% BSA/1 mM EDTA detected in real-time during filtration. (**A**) The filtration process was imaged every minute in order to maximize the number of observed cells despite some cells passing the filter. (**B**) Cells were counted manually during filtration and after the filtration process (*n* = 5). (**C**) Cells were counted using the custom Matlab program using the end-point image and in real-time (*n* = 5).

Cell capture was observed in real-time by fluorescence microscopy throughout the whole filtration process with documentation by either time-serial imaging or video. Four images of the time series are shown in Figure 11A. Using real-time detection, cells that were first captured on the filter, and subsequently passed through the filter after a certain period of time, could be observed and counted. The colored squares mark cells that passed the filter in the following image. Thus, real-time imaging and counting was shown to enable the documentation of those cells. In comparison to quantification of the cells after the complete filtration process using the end-point image, a significantly higher number could be accounted for by real-time detection or video recording. Whereas, the end-point image showed only 63% ± 3% of the spiked cells, 84% ± 4% of the spiked cells could be observed by real-time detection and manual counting (by the help of a handheld clicker) during the filtration process Figure 11B. For automation of the real-time counting, Matlab-based evaluation of the filtration process was used and resulted in cell count of 73% ± 5%, compared to an end-point cell count of 57% ± 7% (Figure 11C).

In comparison to end-point detection of the captured cells after filtration, real-time detection had the advantage that even cells passing the filter after a certain time were taken into account for quantification. Thus, the number of detected cells could be significantly increased, even though not all of the cells could be recovered for further analysis.

## 4. Discussion

In the study presented here, we describe the use of a novel microfluidic device that enables the isolation of 54–89% of target cells spiked into whole blood dependent on their size and deformability. The large breast cancer cells BT-474 could be retained to a higher extent than the smaller colon cancer cells HCT116 or the hematopoietic cells K652. However, still, for the small (diameter approx. 15 µm) HCT116 cells a capture rate of more than 60% was achieved on the filter having a pore size of 7.4 µm. It is not known which cell line represents patient-derived CTCs best, but the employed cell lines were selected to cover cell types with different properties regarding size and deformability. Despite co-enrichment of leukocytes, the pore size of 7.4 µm of the employed TEM-grid was suitable for depletion of approximately 90% of the leukocytes, as determined by optical evaluation of retained leukocytes.

The design of the microfluidic system was optimized in order to obtain a homogenous flow through the filtration area and an even distribution of the cells on the filter. The optimized two-stranded design enhanced the capture rate by 13%. It was further shown that the capture rate in a flow rate range up to 100 µL/s was almost independent of the inlet flow rate and functioned well with pulsatile flow profiles (data shown in the Supplementary material, Figure S4). This suggests that the presented method could be suitable for integration into membrane-based LoC systems such as the *Vivalytic* system, in which pulsatile flow rate profiles are generated.

Our study is the first that comprehensively evaluated the advantage that resides in the possibility to detect cancer cells in real-time during the filtration process and thus increase quantification precision compared to end-point detection of the cells on the filter. Taken together, the filtration set-up presented in this study provides a system for isolation and simultaneous counting of viable CTCs present in a blood sample. Both end-point and real-time evaluation are possible with the advantage of gathering a more complete information of the real number of CTCs present in the sample by real-time analysis regardless of the cells that can be isolated in the filtration process. Keeping in mind that the absolute number of CTCs in the bloodstream is important for therapy monitoring and that the phenotype of the CTCs (size, deformability) might change under therapy, this method improves reproducibility at each time point during serial assessment of the CTC count [36]. Smaller cells, which are able to pass the filter, could still be counted through the real-time detection mode. Hence, they could be observed and taken into the total CTC count, even though the number of finally captured cells might differ between analyses. The real-time detection of cells captured on the filter was performed by a custom Matlab script, which allowed automated evaluation. An improved detection in relation to the end-point determination was achieved, but was lower than the accuracy of manual real-time counting. For this purpose, the cell detection algorithm has to be improved in order to adapt sensitivity and contrast enhancement automatically per frame. In this study, one filter type was chosen for evaluation as the focus relied on evaluating the differences between the end-point and real-time detection method. However, small and deformable cells can still pass the filter despite the pore size of 7.4 µm being smaller than the cells. In order to further improve overall cell retention, filters with different parameters like smaller pores or larger area could be evaluated in addition to real-time detection.

As a next step, the established method will be applied to spike cell numbers of 10 or less cells into 200 µL of whole blood and cells spiked into up to several mL of whole blood and to patient samples with an unknown cell count in order to develop a reproducible diagnostic assay. As real-time detection enables quantification of all target cells present, even very few cancer cells in a small blood volume can be detected. The system is capable of handling up to 2 mL of blood without clogging. However, a large blood volume impedes real-time detection due to high hemoglobin concentration and an overall high leukocyte density. One possibility would be to filter several portions of 200 µL (batch-wise) with a rinsing or cell lysis step step in between.

In the presented method, EpCAM staining was applied as an example for detection of EpCAM-positive CTCs. However, this method can be complemented by a multimarker staining strategy including CK staining for additional detection of cells with heterogeneous EpCAM expression levels or different subclasses of CTCs, for confirmation of the cell type and in order to decrease the probability for counting false positive cells stained due to unspecific antibody binding. For instance, CTCs that underwent EMT that are described as more aggressive [37]. The whole heterogeneity can be covered by staining the cells with an antibody cocktail both unfixed and fixed in order to cover more than one tumor cell-specific marker. Additionally to the CTCs of epithelial origin, cells that underwent EMT could be specifically stained or analyzed at the molecular level. The determination of CTCs including all CTC subtypes could lead to a more complete CTC count considering clinical implications. Furthermore, the capture method provides the possibility to recover cells for downstream analysis, for instance molecular analysis, to increase the information on the disease status and progression obtained from the CTCs of one blood sample for a successful and holistic therapy monitoring at the point-of-care.

## Data Availability

The data that support the findings of this study and the custom scripts written in Matlab used for cell recognition are available from the corresponding authors upon reasonable request.

## Supplementary Materials

The following are available online at https://www.mdpi.com/article/10.3390/bios11090312/s1, Figure S1: SEM image of the TEM-grid used for cell capture, Figure S2: Flow rate profiles used for filtration experiments, Figure S3: Static fluid simulation of two filter holder designs, Figure S4: Capture rate of the HCT116 cells spiked into blood, Figure S5: Filtration set-up mounted on a fluorescence microscope, Figure S6: Recovery rates of cells spiked into PBS, Figure S7: Comparison of identification and correct counting of cell clusters.
