# Supplementary material for: Real-Time Detection of Tumor Cells during Capture on a Filter Element Significantly Enhancing Detection Rate
Source: Biosensors (Basel). 2021 Sep 3;11(9):312. doi: 10.3390/bios11090312 (PMC8472380; doi:10.3390/bios11090312)
Supplement: Supplementary file 1 [file biosensors-11-00312-s001.zip › biosensors-1350631-supplementary.pdf]

Supplementary Material

1. *TEM-Grid as Porous Structure for Filtration*

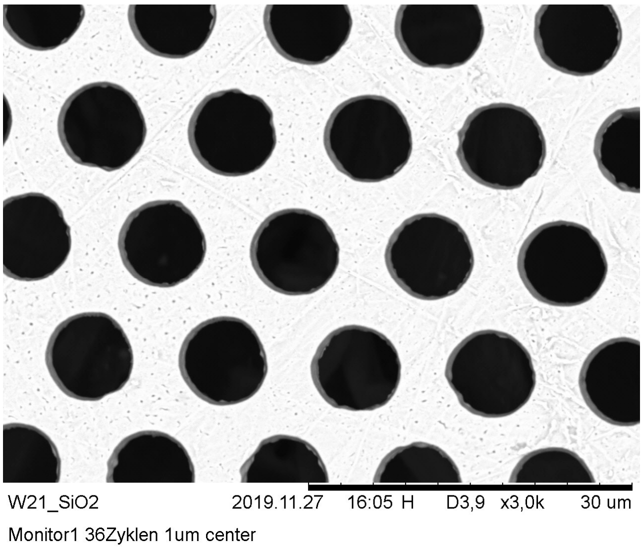

Measurement	Pore size [µm]
**1**	**7.50**
**2**	**7.52**
**3**	**7.48**
**4**	**7.43**
**5**	**7.38**
**6**	**7.34**
**7**	**7.57**
**8**	**7.43**
**9**	**7.43**
**10**	**7.31**
**Mean ± SD**	**7.44 ± 0.06**

Figure S1: SEM image of the TEM-grid used for cell capture. The TEM-grid was analyzed and pore sizes were measured for verification of the specifications. The measurement revealed that the pores were slightly larger than the expected 6.5 µm, and thus porosity was slightly lower.

2. *Flow Rates and Flow Profiles*

With the help of the precisely programmable syringe pump, fluidic control by different flow rates and different flow rate profiles could be examined. The syringe pump was operated either with a continuous or a pulsatile flow profile. With the continuous flow, the influence of different flow rates (2 µL/s, 5 µL/s, 100 µL/s) on the cell retention was investigated. Since this method of cell retention was developed to be compatible with the *Vivalytic* system, the flow rate profiles of this system were mimicked by pulsatile profiles that represent different pumping mechanisms of the system. These pumping mechanisms include (i) pumping with a pump chamber comprising a volume of 30 µL which is actuated through the lower pressure level of the system (Δp = 800 mbar, **Error! Reference source not found.**(b)) and (ii) peristaltic pumping carried out through the pneumatically actuated valves of the system comprising a volume of 1.6 µL (**Error! Reference source not found.** (c)). The resulting flow rate profiles were used in this work and were compared to the filtration process with application of a continuous flow (**Error! Reference source not found.Error! Reference source not found.** (a)).

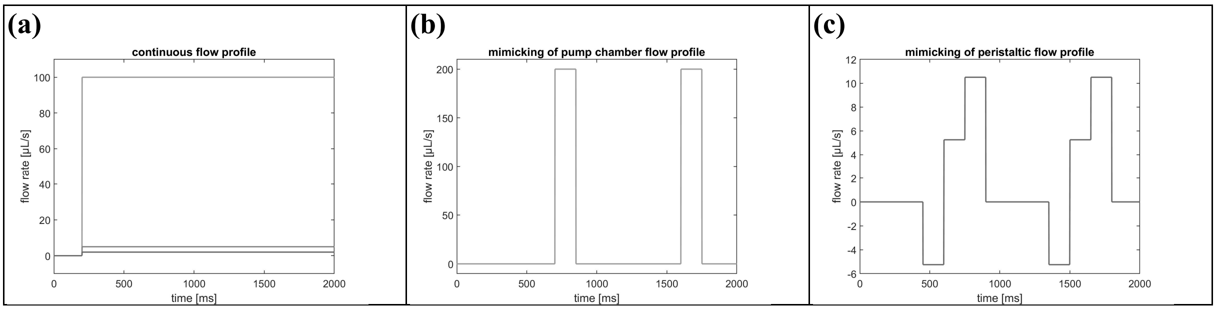

Figure S2: Flow rate profiles used for filtration experiments: (a) Continuous flow with different maximum flow rate (Qmax) of 2 µL/s, 5 µL/s and 100 µL/s. (b) Pulsatile flow profile mimicking fluid transport with a pump chamber comprising a stroke volume of 30 µL. (c) Pulsatile flow profile mimicking fluid transport through three valves comprising a volume of 1.6 µL which are actuated in a peristaltic pattern with a step time of 150 ms.

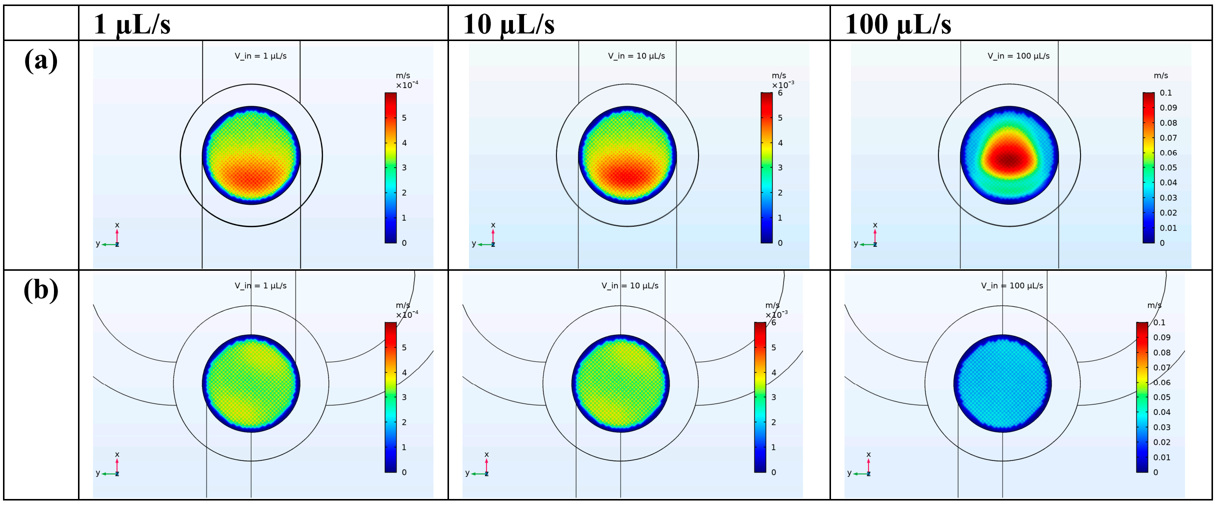

Figure S3: Static fluid simulation of two filter holder designs. Flow through filter area for volume flow rates of 1 µL/s, 10 µL/s and 100 µL/s. **(a)** shows the filter holder design with a single inlet and outlet channel on the top and bottom of the slide. The flow through the filter is not homogeneous and varies over the filter area. In addition, the flowed through area depends on the flow rate at the inlet. **(b)** shows the filter holder design with both the inlet and the outlet divided into two strands. The design features improved flow homogeneity through the filter area, which is independent of the flow rate at the inlet.

3. *Effect of the Flow Rate and Flow Profile on the Capture Rate*

Different continuous flow rates and different flow profiles were applied in the filtration processes in order to mimic the flow conditions on the Vivalytic cartridge. However, as shown in Figure S4 there was no significant difference between the applied flow rates (Figure S4a) and profiles (Figure S4b) leading to the assumption that the filtration unit is suitable for integration into the Vivalytic system and independent of a continuous flow. In addition, the flow rate can be significantly increased to up to 100 µL/s in order to reduce the processing time without adverse effects on the capture rate.

The higher variations of the capture rates at lower flow rates (2 and 5 µL/s) might arise from the increased processing time of the same sample volume compared to 100 µL/s.

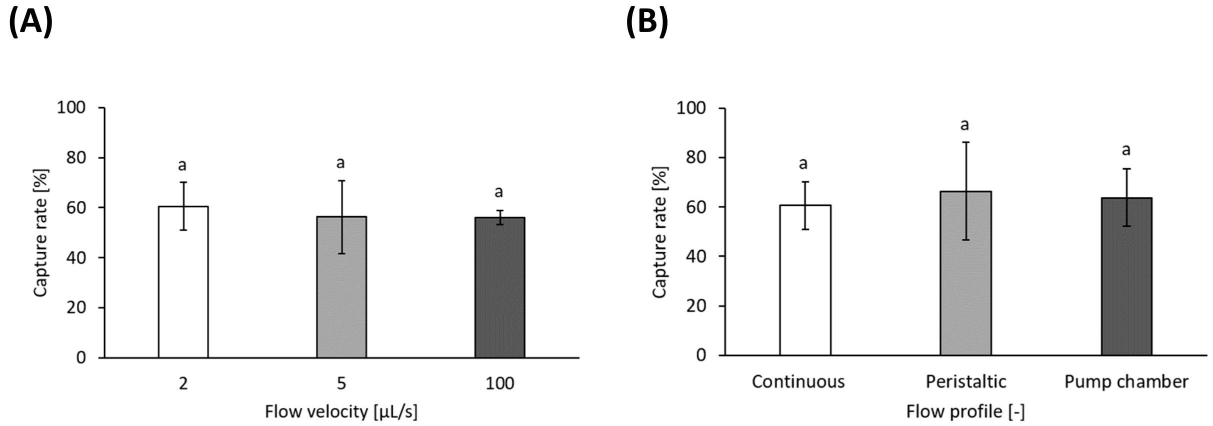

Figure S4: Capture rate of the HCT116 cells spiked into blood was determined dependent on (**A)** flow rates and (**B)** flow profiles. Constant flow rates of 2 (n=10), 5 (n=5) and 100 µL/s (n=3), as well as a continuous flow (n=9), a peristaltic flow (n=9) and a pumping flow (n=9) were compared. No significant differences could be observed ($p < 0.05$).

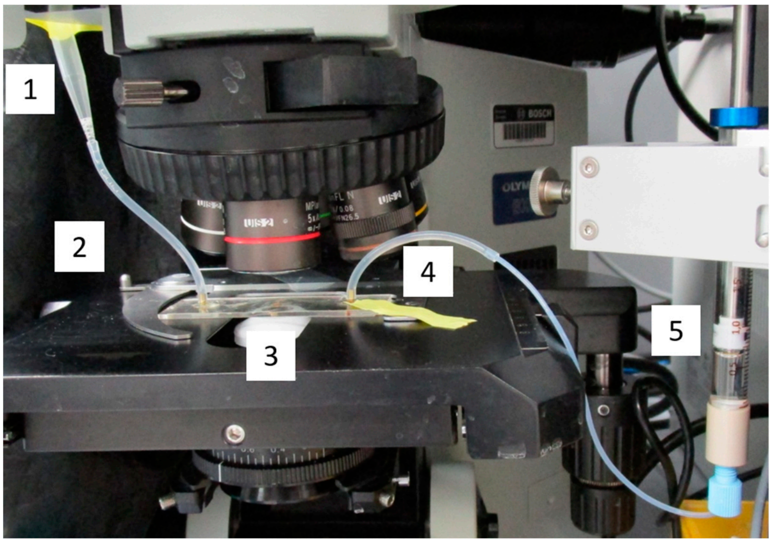

Figure S5: Filtration set-up mounted on a fluorescence microscope.

4. *Preparation/Priming of the Filtration Unit*

System-dependent cell loss was reduced by flushing the set-up with 1 x PBS/1% BSA/ 1 mM EDTA before filtration and was analyzed by processing of a cell suspension using a set-up without integrated filter. Thereby, 100% ± 9% of the cells were recovered. Without this passivation of the system 82% ± 4% were recovered (p-value=0.04, n=3; Supplementary 2).

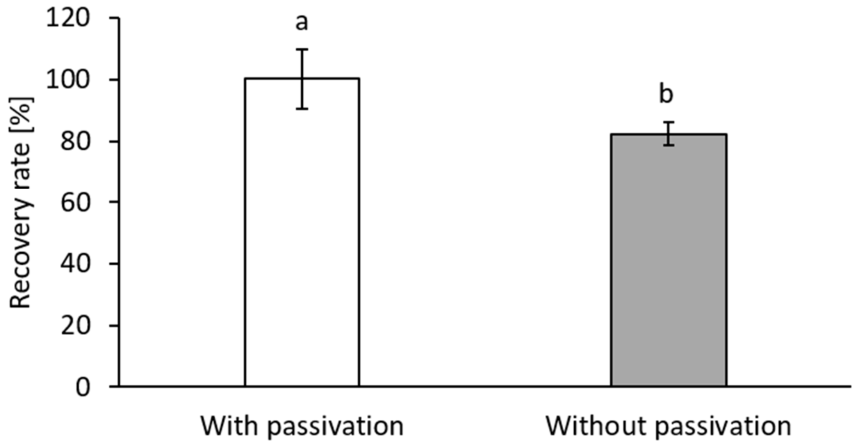

Figure S6: Recovery rates of cells spiked into PBS and drawn through a set-up without filter integrated with and without prior passivation with 1 x PBS/ 1% BSA/ 1mM EDTA. Results are given as means ± SD. Different letters above the bars indicate statistical significance (n=3; $p < 0.05$).

5. *Comparison of the CellSens Dimension and Custom Matlab-based Cell Counting Tool Regarding Cell Cluster Recognition*

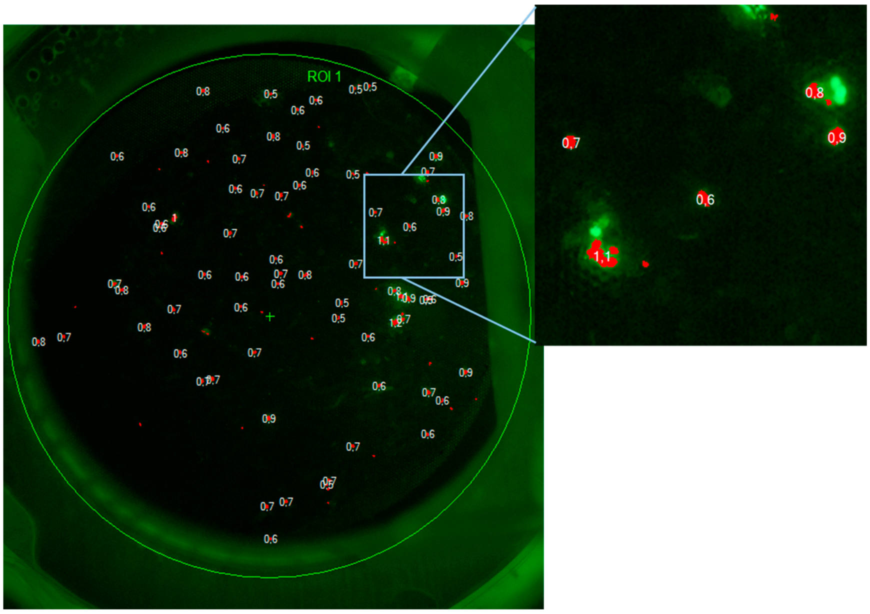

(a)

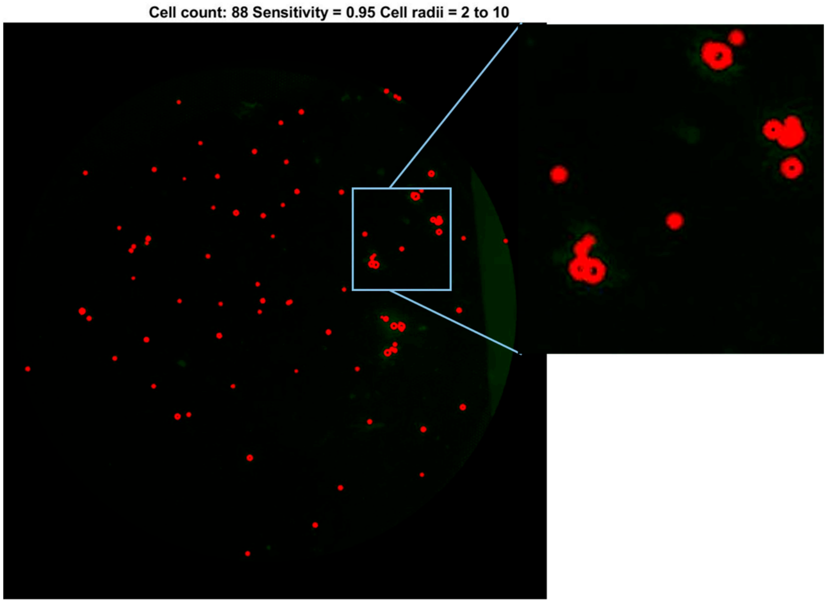

(b)

Figure S7: Comparison of identification and correct counting of cell clusters using (a) CellSens and (b) custom Matlab tool. While cell clusters were often counted as one positive event with the chosen parameters for the CellSens Detection, the established Matlab tool was able to distinguish and separate cells in a cluster more accurately.
